# Polymorphisms of *TGF-β1* and *TGF-β3* in Chinese women with gestational diabetes mellitus

**DOI:** 10.1186/s12884-020-03459-w

**Published:** 2020-12-07

**Authors:** Yinglei Xu, Chunlian Wei, Cuijiao Wu, Mengmeng Han, Jingli Wang, Huabin Hou, Lu Zhang, Shiguo Liu, Ying Chen

**Affiliations:** 1 Department of Medical Genetics, the Affiliated Hospital of Qingdao University, Qingdao, 266000 China; 2 Prenatal Diagnosis Center, the Affiliated Hospital of Qingdao University, Qingdao, 266000 China; 3 Department of Immunology, School of Basic Medical Sciences, Capital Medical University, Beijing, 100000 China; 4 Department of Histology and Embryology, Qingdao University Medical College, Qingdao, 260000 China; 5 Department of Clinical laboratory, the Affiliated Hospital of Qingdao University, Qingdao, China; 6 Department of Endocrinology and Metabolism, the Affiliated Hospital of Qingdao University, Qingdao, 266000 China

**Keywords:** Polymorphism, GDM, PE, TGF-β1, TGF-β3

## Abstract

**Background:**

Gestational diabetes mellitus (GDM) is a pregnancy-specific carbohydrate intolerance Which can cause a large number of perinatal and postpartum complications. The members of Transforming growth factor-β (TGF-β) superfamily play key roles in the homeostasis of pancreatic β-cell and may involve in the development of GDM. This study aimed to explore the association between the polymorphisms of *TGF-β1*, *TGF-β3* and the risk to GDM in Chinese women.

**Methods:**

This study included 919 GDM patients (464 with preeclampsia and 455 without preeclampsia) and 1177 healthy pregnant women. TaqMan allelic discrimination real-Time PCR was used to genotype the TGF-β1 (rs4803455) and TGF-β3 (rs2284792 and rs3917201), The Hardy-Weinberg equilibrium (HWE) was evaluated by chi-square test.

**Results:**

An increased frequency of TGF-β3 rs2284792 AA and AG genotype carriers was founded in GDM patients (AA vs. AG + GG: χ^2^ = 6.314, *P* = 0.012, OR = 1.270, 95%CI 1.054–1.530; AG vs. GG + AA: χ^2^ = 8.545, *P* = 0.003, OR = 0.773, 95%CI 0.650–0.919). But there were no significant differences in the distribution of TGF-β1 rs4803455 and TGF-β3 rs3917201 between GDM and healthy women. In addition, no significant differences were found in allele and genotype frequencies among GDM patients with preeclampsia (PE).

**Conclusions:**

The AA and AG genotype of TGF-β3 rs2284792 polymorphism may be significantly associated with increased risk of GDM in Chinese population.

**Supplementary Information:**

The online version contains supplementary material available at 10.1186/s12884-020-03459-w.

## Background

GDM is the most common maternal metabolic disturbance that is defined as glucose intolerance of variable severity with onset or first detection during pregnancy [1, 2]. The prevalence of GDM varies from 1 to 22% of all pregnancies depending on different populations and diagnostic criteria [3–5]. GDM not only increases the risk of maternal and fetal perinatal complications, but also has long-term adverse consequences for offspring [6, 7]. The most familiar complication following GDM is PE which shares common clinical risk factors with GDM such as obesity, advanced maternal age and diabetes [8]. GDM is characterized by increased insulin resistance and defective insulin secretion which is due to the inability of pancreatic β cells [2]. However, the etiology is complex due to disordered metabolism and intrauterine environment during pregnancy. Extensive efforts have been made to explore the pathogenesis and to find new targets for prediction of GDM [2, 9, 10].

The TGF-β superfamily, including TGF-β isoforms, activins, inhibins and bone morphogenetic proteins (BMPs), is involved in a myriad of biological processes such as cell proliferation, differentiation and death [11]. In addition, TGF-β signaling has been indicated to play key roles in the development of GDM and GDM risk factors. BMPs disfunction will impair insulin signal and glucose homeostasis in the setting of diabetes [12]. Activins can promote the proliferation of pancreatic β-cell and secretion of insulin [13]. TGF-β isoforms are known to stimulate adipocyte proliferation, insulin resistance and subclinical inflammation [14].

In recent years, the role of genetic factors in the pathogenesis of GDM has been increasingly investigated. The major genetic studies of GDM are candidate gene studies, which have revealed that some single nucleotide polymorphisms (SNPs) in cytokine genes are associated with susceptibility to GDM [15, 16]. SNPs within the coding and signal sequences can affect gene transcriptional activity, and then change the production of proteins [17]. Several studies have reported that altered cytokines expression are related to the severity and progression of the GDM [18, 19]. Therefore, the cytokine genes with positive SNP loci may be a pregnancy biomarker for screening GDM.

TGF-β1 and TGF-β3 belong to TGF-β isoforms and have differential expression in the human endometrium and placenta [11]. Both of them contribute to normal homeostasis of pancreas and insulin action [20]. The enhanced expression of TGF-β1 induced by hyperglycemia was detected in individuals with GDM [21, 22]. Although there is no direct relation between TGF-β3 and GDM, TGF-β3 participates in many GDM complications such as PE and pregnancy-induced hypertension [23]. Three tag SNPs (rs4803455, rs2284792, and rs3917201), located in introns of *TGF-β1* and *TGF-β3* locus respectively, can affect the transcriptional activity and change the expression of proteins [24–26]. Therefore, we supposed that these three SNPs might be target SNPs, and try to investigate the relationship between polymorphisms of *TGF-β1*, *TGF-β3* and the risk of GDM.

## Methods

### Subjects

This study was conducted based on 919 pregnant women with GDM and 1177 healthy pregnant women with normal glucose tolerance, recruited from the clinical pregnancy registries at the Affiliated Hospital of Qingdao University, People’s Hospital of Liaocheng City and People’s Hospital of Linyi City. Informed consent was issued and signed by all subjects and all investigations were approved by the ethics committee of the Affiliated Hospital of Qingdao University.

All the participants underwent a 75 g oral glucose tolerance test (OGTT) at 24–28 weeks’ gestation. The diagnosis of GDM was based on the International Association of Diabetes and Pregnancy Study Groups (IADPSG) criteria when one of the following plasma glucose values in the OGTT was met or exceeded, fasting plasma glucose 92 mg/dl (5.1 mmol/l), 1 h plasma glucose 180 mg/dl (10.0 mmol/l) and 2 h plasma glucose 153 mg/dl (8.5 mmol/l). Plasma glucose during OGTT of the follow-up study was measured by enzymatic hexokinase photometric assay. Exclusion criteria included heart diseases, chronic hypertension, diabetes mellitus, thyroid diseases, kidney disorders, abnormal liver function, twin or multiple pregnancies, as well as in-vitro fertilization in the present gestation. Women were recruited in the study group when first diagnosed as GDM at 24–28 weeks’ gestation. Then, they were taken blood for testing before diet control and insulin therapy. Besides, 919 GDM patients were categorized into 455 without PE and 464 with PE which was determined on the base of the questionnaire, clinical features, and data. A newly onset of hypertension (≥140/90 mmHg) with proteinuria C of 300 mg or higher in 24-h after 20 weeks of gestation was diagnosed as PE.

## Methods

Genomic DNA was extracted from peripheral venous blood by alkaline lysis method and collected by centrifugal column in the Qiagen blood DNA extraction kit (Qiagen, Hilden, Germany). TaqMan allelic discrimination real-time PCR (Life Technologies, Grand Island, NY, USA) was used to genotype the polymorphisms of rs4803455 in *TGF-β1*, rs2284792 and rs3917201 in *TGF-β3*. The TaqMan probes and primers were designed by Applied Bio-systems or Life Technologies (New York. USA). *TGF-β1* and *TGF-β3* were amplified using the following primers: 5′-GCTGCAAACATTCTGGGGTTT-3′ for TGF-β1 rs4803455, 5′-GGGTGGGACCAGGGAATCT-3′ for TGF-β3 rs2284792 and 5′-CGCCTCAAGAAGCAGAAGGAT-3′ for TGF-β3 rs3917201. Reaction volume was 25 μl: 1.25 μL 20 × SNP Genotyping Assay, 12.5 μL 2 × PCR Master Mix, and 11.25 μL DNA and DNase-free water. 1000™ Thermal cycler and CFX96™ Real-time system (Bio-Rad, California, USA) were carried out to amplifications as following conditions: 95 °C for 3 min, followed by 45 cycles at 95 °C for 15 s and 60 °C for 1 min. The fluorescent signals from VIC/FAM-labeled probes were detected for each cycle. Discrimination of genotypes was conducted with BioRad CFX manager 3.0 software.

### Statistical analysis

Statistical software package IBM SPSS 22.0 (SPSS Inc., Chicago, IL, USA) was used to manipulate all data. Student’s *t*-test was utilized to compare the demographic and clinical characteristics of cases and controls. An analysis of variance (ANOVA) was used to conduct the genotype-phenotype analysis. A chi-square test was performed to assess the HWE in the controls. Allelic and genotypic distributions were enrolled in the comparison by using Pearson’s χ^2^ test which was substituted with Fisher’s exact test when expected values were below 5. *P* <  0.05 (two-sided) was considered to represent statistically significance. Odds ratios (ORs) and 95% confidence intervals (CIs) were used to reveal the relative risk degree. A *P*-value < 0.05 (two-sided) was taken as statistical significance for all statistical analyses.

## Results

### Demographic and clinical characteristics of GDM and controls

Subjects were categorized into 919 GDM patients and 1177 controls. Demographic and clinical data of different groups were summarized in the supplemental table.

Both groups had similar age distribution, times of gravidity, and number of abortions. The mean age of cases and controls was 30.71 ± 4.18 and 30.75 ± 4.21 years old. However, in GDM group, weeks of admission and delivery intended to be earlier (*P* <  0.001) and the weight gain of newborns was heavier than in the control group as expected (*P* <  0.001).

### *TGF-β1* and *TGF-β3* polymorphism analysis

The subjects of the control group enrolled in this study were in accordance with HWE for these SNPs and displayed a group representative at the significance level of P>0.05.

The distributions of the genotypes and alleles in GDM cases and controls were reported in Table 1. We observed a statistically significant difference between GDM and healthy women in the frequencies of *TGF-β3* rs2284792 (χ^2^ = 9.064, *P* = 0.011). However, no statistical differences were detected either in *TGF-β1* rs4803455 or in *TGF-β3* rs3917201 between two groups in terms of genotypic frequencies. As shown in Table 1, the allelic frequencies of rs2284792 between two groups were not obviously different (χ^2^ = 1.592, *P* = 0.207, OR = 1.082, 95%CI 0.957–1.224). When categorized into three models (AA vs AG + GG, GG vs AG + AA and AG vs GG + AA), there was a significant difference between these two groups (For AA vs AG + GG model, χ^2^ = 6.314, *P* = 0.012, OR 1.270, 95%CI 1.054–1.530; For AG vs GG + AA model, χ^2^ = 8.545, *P* = 0.003, OR = 0.773, 95%CI 0.650–0.919). Consistently, allelic frequencies of TGF-β1 rs4803455 or TGF-β3 rs3917201 were statistically insignificant.

**Table 1:** The comparison of genotypic and allelic frequencies of all SNPs between GDM (all cases) and controls

	Cases	Controls	***χ2***	***p***-value	OR	95%CI
rs4803455						
Genotypes						
AA	142	173	1.206	0.574		
AC	412	556				
CC	365	448				
Alleles						
A	696	902	0.089	0.766	1.019	0.899–1.156
C	1142	1452				
rs2284792						
Genotypes						
AA	268	404	9.064	0.011*		
AG	487	548				
GG	164	225				
AA	268	404				
AG + GG	651	773	6.314	0.012*	1.270	1.054–1.530
GG	164	225				
AG + AA	755	952	0.511	0.458	1.088	0.871–1.360
AG	487	548				
GG + AA	432	629	8.545	0.003*	0.773	0.650–0.919
Alleles						
A	1023	1356	1.592	0.207	1.082	0.957–1.224
G	815	998				
rs3917201						
Genotypes						
GG	220	294	0.303	0.895		
AG	468	592				
AA	231	291				
Alleles						
A	908	1180	0.218	0.641	1.029	0.911–1.163
G	930	1174				

*p < 0.05 is considered statistically significant, *OR* ODDs ratio, *CI* Confidence interval

### *TGF-β1* and *TGF-β3* polymorphism analysis between GDM patients with and without PE

To further study the association between variants of the three SNPs and complications, samples were categorized into GDM cases with and without PE. The distributions of the genotypes and alleles in GDM patients with and without PE are shown in Tables 2 and 3.

**Table 2:** The comparison of genotypic and allelic frequencies of all SNPs between GDM without PE and controls

	Cases	Controls	***χ2***	***p***-value	OR	95%CI
rs4803455						
Genotypes						
AA	70	173	0.631	0.730		
AC	205	556				
CC	180	448				
Alleles						
A	345	902	0.046	0.831	1.017	0.869–1.191
C	565	1452				
rs2284792						
Genotypes						
AA	122	404	9.774	0.008*		
AG	247	548				
GG	86	225				
AA	122	404				
AG + GG	333	773	8.476	0.004*	1.472	1.122–1.813
GG	86	225				
AG + AA	369	952	0.010	0.921	1.014	0.769–1.336
AG	247	548				
GG + AA	208	629	7.842	0.005*	0.734	0.590–0.912
Alleles						
A	491	1356	3.555	0.059	1.159	0.994–1.352
G	419	998				
rs3917201						
Genotypes						
GG	108	294	0.571	0.752		
AG	227	592				
AA	120	291				
Alleles						
A	443	1180	0.549	0.459	1.060	0.909–1.235
G	467	1174				

*p < 0.05 is considered statistically significant, *OR* ODDs ratio, *CI* Confidence interval

**Table 3:** The comparison of genotypic and allelic frequencies of all SNPs between GDM with PE and controls

	Cases	Controls	***χ2***	***p***-value	OR	95%CI
rs4803455						
Genotypes						
AA	72	173	1.266	0.531		
AC	207	556				
CC	185	448				
Alleles						
A	351	902				
C	577	1452				
rs2284792						
Genotypes						
AA	146	404	3.619	0.164		
AG	240	548				
GG	78	225				
Alleles						
A	532	1356	0.021	0.885	1.011	0.867–1.179
G	396	998				
rs3917201						
Genotypes						
GG	112	294	0.359	0.836		
AG	241	592				
AA	111	291				
Alleles						
A	463	1180	0.015	0.903	1.009	0.867–1.175
G	465	1174				

*p < 0.05 is considered statistically significant, *OR* ODDs ratio, *CI* Confidence interval

In GDM cases without PE group, the statistical difference between cases and controls in genotypic distributions of TGF-β3 rs2284792 was observed (χ^2^ = 9.774, *P* = 0.008). Also, the same was found for allelic frequencies in AA vs AG + GG (χ^2^ = 8.476, *P* = 0.004 OR = 1.427, 95%CI 1.122–1.813) and AG vs GG + AA (χ^2^ = 7.842, *P* = 0.005 OR = 0.734, 95%CI 0.590–0.912). In contrast to TGF-β3 rs2284792, no obvious difference was found in either the genotypic distributions or allelic frequencies of *TGF-β1* rs4803455 and TGF-β3 rs3917201 among GDM only cases.

In GDM cases with PE group, however, no obvious difference was found in either the genotypic distributions or allelic frequencies of three SNPs (for rs4803455, χ^2^ = 1.266, *P* = 0.531 by genotype, χ^2^ = 0.069, *P* = 0.793, OR = 1.021, 95%CI 0.873–1.194 by allele; when for rs2284792, χ^2^ = 3.619, *P* = 0.164 by genotype, χ^2^ = 0.021, *P* = 0.885, OR = 1.011, 95%CI 0.867–1.1791by allele; and for rs3917201, χ^2^ = 0.359, *P* = 0.836 by genotype, χ^2^ = 0.015, *P* = 0.903, OR = 1.009, 95%CI 0.867–1.175 by allele).

### Analysis of genotype-phenotype relationship

Analysis of the relationship between the genotypes of TGF-β3 rs2284792 and demographic characteristics among total GDM patients was shown in Table 4. However, no statistical differences were found for the genotype-phenotype relationship of rs2284792.

**Table 4:** Associations between genotypes of rs2284792 and characteristics among total GDM patients

Rs2284792(A/G)	AA	AG	GG	AA vs. AG	AA vs GG	AG vs GG	AA vs AG + GG	GG vs AG + AA	AG vs GG + AA
	(n)	(n)	(n)	_***P***_a	_***p***_b	_***p***_c	_***p***_d	_***p***_e	_***p***_f
Cases	268	487	164						
Demographic characteristics (Mean ± S)									
Fasting blood glucose (mmol/l)	5.90 ± 2.13	5.75 ± 2.22	5.76 ± 2.33	0.087	0.097	0.960	0.059	0.407	0.360
Systolic blood pressure (mmHg)	137.65 ± 22.24	137.41 ± 24.86	137.53 ± 22.06	0.166	0.425	0.468	0.224	0.997	0.231
Diastolic blood pressure (mmHg)	77.09 ± 12.36	77.17 ± 11.86	77.01	0.519	0.560	0.137	0.972	0.186	0.177
WBC (× 10^9^/L)	10.51 ± 2.99	10.62 ± 3.05	10.62 ± 3.05	0.122	0.144	0.921	0.099	0.514	0.436
RBC (× 10^12^/L)	4.41 ± 1.73	4.37 ± 1.17	4.51 ± 1.91	0.588	0.233	0.234	0.997	0.085	0.185
Hb (g/L)	116.43 ± 17.40	116.47 ± 14.12	116.32 ± 14.31	0.823	0.451	0.308	0.753	0.343	0.560
neutrophil (×10^9^/L)	8.40 ± 2.54	8.24 ± 2.43	8.44 ± 2.81	0.157	0.779	0.068	0.557	0.167	0.056
PLT (×10^9^/L)	227.30 ± 58.26	226.72 ± 67.55	226.69 ± 58.86	0.102	0.149	0.953	0.079	0.562	0.252
PT (s)	10.64 ± 1.59	10.68 ± 1.60	10.71 ± 1.83	0.436	0.283	0.572	0.330	0.402	0.808
APTT (s)	30.57 ± 3.93	30.52 ± 3.91	30.63 ± 3.38	0.441	0.446	0.134	0.720	0.192	0.300
ALT (IU/L)	27.66 ± 21.05	26.95 ± 16.47	27.14 ± 19.28	0.065	0.356	0.663	0.075	0.920	0.132
AST (IU/L)	29.82 ± 18.52	29.53 ± 16.51	29.57 ± 19.35	0.384	0.599	0.930	0.389	0.871	0.510
Creatinine (umol/L)	58.58 ± 18.75	58.49 ± 19.15	58.49 ± 17.09	0.716	0.788	0.986	0.704	0.931	0.782
Body mass before pregnancy (kg)	59.73 ± 3.43	63.58 ± 0.95	63.04 ± 0.74	0.146	0.159	0.647	0.116	0.959	0.422
Body mass increase during pregnancy (kg)	17.53 ± 1.25	17.57 ± 0.62	16.24 ± 0.47	0.978	0.318	0.086	0.614	0.072	0.123
BMI before pregnancy (kg/m^2^)	23.97 ± 0.79	24.30 ± 0.34	24.23 ± 0.40	0.698	0.804	0.896	0.754	0.974	0.837
BMI at birth (kg/m^2^)	30.7 ± 0.92	30.91 ± 0.42	30.43 ± 0.46	0.841	0.827	0.456	0.964	0.463	0.472

*P*^a^ value between AA and AG; *P*^b^ value between AA and GG; *P*^c^ value between AG and GG; *P*^d^ value between **AA and AG + GG**; *P*^e^ value between **GG and AG + AA;** *P*^f^ value between **AG and GG + AA**

*p* < 0.05 is considered statistically significant. *WBC* White Blood Cell, *RBC* Red Blood Cell, *Hb* Hemoglobin, *PLT* Platelet, *PT* prothrombin time, *APTT* activated partial thromboplastin time, *ALT* glutamic pyruvic transaminase, *AST* glutamic oxaloacetic transaminase

## Discussion

In this study, the associations between *TGF-β1*, *TGF-β3* polymorphisms and GDM were examined in a Chinese population. Among women with GDM, we firstly found an effective association between the tag SNP TGF-β3 rs2284792 and GDM risk. Besides, we confirmed that the A allele and the AA and AG genotypes were susceptible, while the G allele/GG genotype may be protective factors. However, there were no statistically significant differences in the distribution of TGF-β1 rs4803455 and TGF-β3 rs3917201 genotypes between GDM and healthy women.

Previous genetic study of GDM is to find candidate genes that was based on biological plausibility [27]. Recently, genome-wide association analysis studies were performed to identify some susceptibility genes associated with GDM [4]. The genetic variants of candidate genes have been revealed to contribute to the risk of GDM. For example, rs12255372 variant in *Transcription factor 7-like 2* was indicated to interact with adiposity to alter β-cell function in 132 Mexican-American families with GDM [28]. The homozygosity for *G972R* polymorphism in *Insulin receptor substrate-1* might indicate an increased risk for GDM in Saudi women [29]. There was also was significantly associated with genotypes and alleles of the CC chemokine ligand 2 rs1024611 and rs4586 polymorphisms [18] and GDM. Interestingly, many GDM associated candidate genes can express cytokines implicated in the inflammatory conditions during pregnancy [30].

GDM is characterized by varying degrees of hyperglycemia due to the inability of pancreatic β-cells to adequately respond to the increased insulin requirements during the second and third trimester [31, 32]. The etiology of GDM may be explained by many factors including cytokines, hormones, lifestyle as well as genetic disposition [33]. TGF-β isoforms are multifunctional factors that regulate embryonic development, immunity, and epithelial homeostasis [34]. Genetic polymorphisms of TGF-β isoforms were linked with an increased likelihood of having GDM and complications such as PE and diabetic nephropathy [15, 35]. With such attributes, we chose *TGF-β1* and *TGF-β3* as target genes to uncover the genetic disposition of GDM.

TGF-β1 is reported to be a key cytokine in insulin resistance and obesity. Over-expression of TGF-β1 can lead to decreased β-cell mass and insulin secretion [36]. TGF-β1 rs4803455 polymorphism is an A/C single-nucleotide variation on chromosome 19q13.2 and can alter the expression of insulin receptor substrate 2 associated with insulin resistant in GDM, but not depending on its expression in the pathway [37]. Moreover, a previous study suggested that TGF-β1 rs4803455 showed the effectiveness to capture the associations with cancer risk [38]. However, our data revealed that TGF-β1 rs4803455 was not a significant risk factor of GDM in the Chinese Population. The difference between these studies could be attributed to the discordance of population genetic background. However, the finite sample size in these studies is another limiting factor to have a coincident conclusion.

This is the first study to show the relationship between the genetic polymorphism of *TGF-β3* gene and GDM. Candidate SNPs previously described were chosen based on their location within the gene, and a tag SNP (rs2284792: A > G) selected with SNP picker using data from the Caucasian population was located within the introns of *TGF-β3* [39]. Our studies revealed an effective association between the tag SNP rs2284792 and GDM risk. Besides, we confirmed that the A allele and the A allele-containing genotypes (AA and AG) were susceptible, while the G allele/GG genotype may be protective factors. TGF-βs in mammals exhibit many overlapping biological activities and appear interchangeable. TGF-β3 knock-in ameliorate inflammation due to TGF-β1 deficiency while promoting glucose tolerance [40]. Reduced TGF-β3 expression can cause hypertrophy and induce glucose intolerance [41]. Therefore, altered generation made by polymorphic variants in *TGF-β3* may affect glucose homeostasis, thus leading to GDM.

GDM is a transient presentation of long-standing metabolic malfunction and may be expected to have an association with PE [42]. The pathophysiology of PE is characterized by endothelial dysfunction which may be induced by down-regulation of TGF-β signaling. TGF-β isoforms were predisposed to have obvious susceptible associations with PE and were supposed as a biomarker for assessment of PE severity [43, 44]. *TGF-β1* codon 10 T/C was observed to have a higher frequency of T>C allele in Type 2 Diabetes Mellitus patients with hypertension [45]. A fetal *TGF-β3* variant (rs11466414) is associated with PE in a predominantly Hispanic population [44]. In consideration of comparable clinical characteristics, we hypothesized that the variants of TGF-β isoforms may relate to the development of both disease conditions. Then, we analyzed *TGF-β1* (rs4803455) and *TGF-β3* (rs2284792 and rs3917201) polymorphisms among GDM cases with PE. However, no obvious difference was found in either the genotypic distributions or allelic frequencies among above three SNPs. The complexity of several pathogenic pathways including metabolic, immune, and endothelial dysfunction can account for the invalid assumption. Insulin resistance which is highly prevalent in patients with GDM can only partially explain the development of PE [42]. To sum up, TGF-β3 rs2284792 may be the independent effective genetic locus for GDM alone.

## Conclusions

This study indicated that the AA and AG genotype rs2284792 polymorphism of *TGF-β3* was associated with the increased risk of GDM. However, some evident shortcomings are the limited sample size and the different ethnic origins. Furthermore, some environmental factors, such as behavioral and pharmacological interventions, will be considered in our future studies. All these studies highlight the need of long-term cohort studies of women with GDM for ultimately improving pregnancy outcomes.

## Supplementary Information

**Additional file 1: Table S1**. The demographic and clinical characteristics of GDM and controls.

## Data Availability

The original data used to support the findings of this study are available from the corresponding author upon request.

## Abbreviations

TGF-β: Transforming growth factor-β
GDM: Gestational diabetes mellitus
HWE: Hardy-Weinberg equilibrium
PE: Preeclampsia
SNPs: Signal Nucleotide Polymorphisms
OGTT: Oral glucose tolerance test
ORs: Odds ratios
Cis: confidence intervals
