# Supplementary material for: Polymorphisms of TGF-β1 and TGF-β3 in Chinese women with gestational diabetes mellitus
Source: BMC Pregnancy Childbirth. 2020 Dec 7;20:759. doi: 10.1186/s12884-020-03459-w (PMC7720537; doi:10.1186/s12884-020-03459-w)
Supplement: Supplementary file 1 — Additional file 1: Table S1. The demographic and clinical characteristics of GDM and controls. [file 12884_2020_3459_MOESM1_ESM.docx]

**Supplemental Table** The demographic and clinical characteristics of GDM and controls

| **Characteristics** | GDM (N=919) | Controls (N=1177) | *t* | *P* |
| --- | --- | --- | --- | --- |
| Maternal age(years) | 30.71±4.18 | 30.75±4.21 | 1.366 | 0.971 |
| Gestational age at admission (weeks) | 37.81±2.67 | 39.23±1.30 | 102.532 | ＜0.001 |
| Childbirth (weeks pregnant) | 37.05±2.74 | 39.37±1.21 | 162.526 | ＜0.001 |
| Current number of gravidity | 2.91±1.28 | 2.87±1.33 | -0.963 | 0.336 |
| Number of abortion | 1.84±0.92 | 1.83±1.00 | -0.226 | 0.821 |
| Weight fetal (g) | 3712.10±450.01 | 3437.28±332.57 | -964.013 | ＜0.001 |

p < 0.05 is considered statistically significant
